# Synthesis, Characterization, Electrochemistry, Photoluminescence and Magnetic Properties of a Dinuclear Erbium(III)-Containing Monolacunary Dawson-Type Tungstophosphate: [{Er(H_2_O)(CH_3_COO)(P_2_W_17_O_61_)}_2_]^16−^

**DOI:** 10.3390/molecules25184229

**Published:** 2020-09-15

**Authors:** Masooma Ibrahim, Ananya Baksi, Yan Peng, Firas Khalil Al-Zeidaneen, Israël M. Mbomekallé, Pedro de Oliveira, Christopher E. Anson

**Affiliations:** 1 Institute of Nanotechnology, Karlsruhe Institute of Technology, Hermann von-Helmholtz Platz 1, 76344 Eggenstein-Leopoldshafen, Germany; ananya.baksi@kit.edu; 2 Institute of Inorganic Chemistry, Karlsruhe Institute of Technology, Engesserstrasse 15, 76131 Karlsruhe, Germany; Py1688@yahoo.com (Y.P.); firas.al-zeidaneen@kit.edu (F.K.A.-Z.); Christopher.Anson@kit.edu (C.E.A.); 3 Equipe d’Electrochimie et de Photo-électrochimie, Institut de Chimie Physique, Université Paris-Saclay, UMR 8000 CNRS, F-91405 Orsay, France; israel.mbomekalle@universite-paris-saclay.fr (I.M.M.); pedro.almeida-de-oliveira@universite-paris-saclay.fr (P.d.O.)

**Keywords:** polyoxometalates, catalysis, mass spectrometry, molecular magnetism, electrochemistry, lanthanide, erbium

## Abstract

Reaction of the trilacunary Wells−Dawson anion {α-P_2_W_15_O_56_}^12−^ with Er^III^ ion in a 1 M LiOAc/HOAc buffer (pH 4.8) solution produces a dinuclear erbium(III) substituted sandwich-type structure [{Er(H_2_O)(CH_3_COO)(P_2_W_17_O_61_)}_2_]^16−^ (**1**). The isolated compound was structurally characterized using single crystal and powder X-ray diffraction, FTIR spectroscopy, mass spectrometry and thermogravimetric analysis. The electrochemical, electrocatalytic, photoluminescence and magnetic properties of **1** were investigated.

## 1. Introduction

Polyoxometalates (POMs) are an emerging class of molecular metal oxides, typically comprised of early transition metals (such as W^6+^, Mo^6+^, V^5+^, Nb^5+^ and Ta^5+^) in their high oxidation states and are generally constructed from the connections of {MO_x_} polyhedra through edge-, corner- or face-sharing linkages. The intrinsic chemical behavior of POMs such as acidity and redox properties can be tuned by the insertion of metal cations (e.g., d- and f-block elements) into anionic POM frameworks. Lacunary POM species, which can be derived by removal of (M = O)^n+^ groups from Keggin or Wells–Dawson POM skeleton (e.g., {PW_11_}, {SiW_9_}, {P_2_W_17_}, {P_2_W_15_}), can act as all inorganic ligands with various metal cations resulting in functional molecular systems with an unmatched and tunable range of physical and chemical properties [1,2,3,4,5]. Focusing only on Ln-containing POMs (Ln-POMs); lacunary POMs with nucleophilic oxygen centers are ideal multidentate ligands for lanthanide ions owing to their high oxophilic nature. In addition, Ln ions can have high coordination numbers, thus they can link POM building blocks to create discrete giant Ln-POM structures or polymeric networks (1D, 2D and 3D extended POMs) [6,7,8,9,10]. However, in Ln-POMs due to their larger sizes and consequently larger coordination numbers compared to 3d metal ions, the formation of lanthanide-oxo-hydroxo clusters within the POM framework has been rarely observed [9,11,12]. Until now, there are only a few reports on the sandwich-type Ln-POM family, where lanthanide metal centers (Ln^III^ nuclearity ≥ 2) are sandwiched between two lacunary POM ligands [13,14,15,16,17,18,19,20,21,22,23,24,25,26]. Lanthanide-containing POM assemblies have demonstrated outstanding properties in various fields of research such as molecular magnetism [27], imaging [28], photoluminescence [29], catalysis [30,31] and electrochemistry [32]. Magnetic POMs have attracted an increasing interest after the discovery of the first POM-based single molecule magnet (SMM) [ErW_10_O_36_]^9–^ [33]. In the field of nanomagnetism, the diamagnetic POMs as bulky inorganic ligands could be favorable in minimizing intermolecular magnetic coupling (i.e., to behave as an effective magnetic isolator between the neighboring molecules for magnetic dilution purposes) [27,34]. Some of the important intrinsic properties of POM clusters are their thermal and hydrolytic stability, tunability of acid and redox properties and high solubility in a variety of solvents, which make them promising catalysts for different chemical processes. The Ln-POMs have exhibited noteworthy catalytic performances in various chemical processes [30,31,32]. The photochemistry of Ln-POMs is also of great interest; POM ligands usually act as light harvesting antennae in photoluminescent lanthanopolyoxotungstate species, which sensitize Ln^III^ centers (such as Sm^III^, Eu^III^, Tb^III^ and Dy^III^) by absorbing incident light and then transferring this excitation to Ln^III^ ions through an energy transfer process [29,35].

As part of our ongoing research on Ln-POM chemistry, we herein report the synthesis of dinuclear Er^III^-containing 34-tungsto-4-phosphate (v) [{Er(H_2_O)(CH_3_COO)(P_2_W_17_O_61_)}_2_]^16−^ (**1**), which has been obtained under normal bench conditions by the reaction of Er(NO_3_)_3_^.^6H_2_O and trilacunary POM ligand Na_12_[α-P_2_W_15_O_56_]^.^18H_2_O {P_2_W_15_} in 1 M LiOAc buffer pH 4.8 and its characterization by single crystal X-ray crystallography (SCXD), powder X-ray diffraction (PXRD), Fourier-transform infrared (FTIR) spectroscopy, elemental analysis, thermogravimetric analysis, mass spectrometry, UV—vis absorption spectroscopy and luminescence spectroscopy. Additionally, electrochemical and magnetic properties were also studied.

## 2. Results and Discussion

### 2.1. Synthesis

The title polyanion [{Er(H_2_O)(CH_3_COO)(P_2_W_17_O_61_)}_2_]^16−^ (**1**) was prepared by reaction of the Er(III) ion with the trilacunary Dawson-type polyanion [α-P_2_W_15_O_56_]^12−^ in 1 M LiAOc buffer (pH 4.8) media at 80 °C. The isolation of **1** requires the LiOAc/HOAc buffer (pH 4.8) solution as the synthetic medium.

### 2.2. Single-Crystal X-Ray Structure Determination

**Figure 1:**
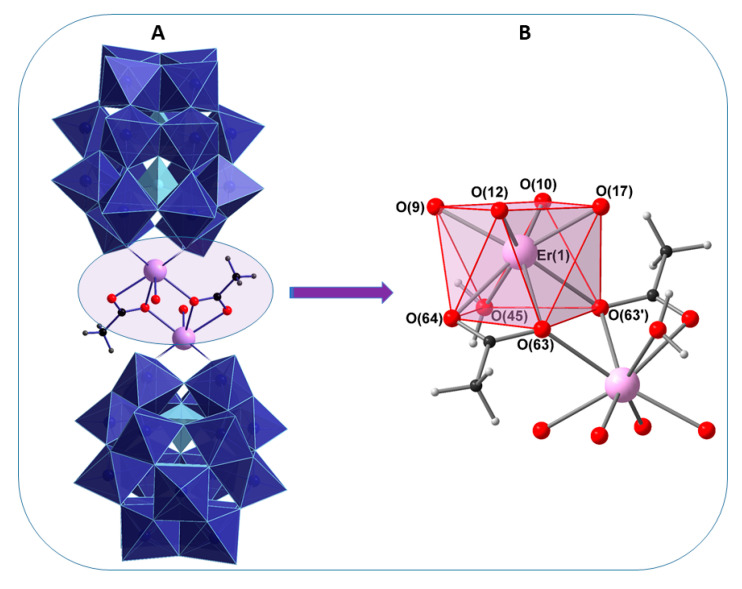
Combined polyhedral (**A**)/ball-and-stick (**B**) representation of **1** and the Er_2_ core. Color code: WO_6_ blue octahedra, PO_4_ sky blue tetrahedra, Er lavender, O red, C black, H small gray spheres.

**Figure 2:**
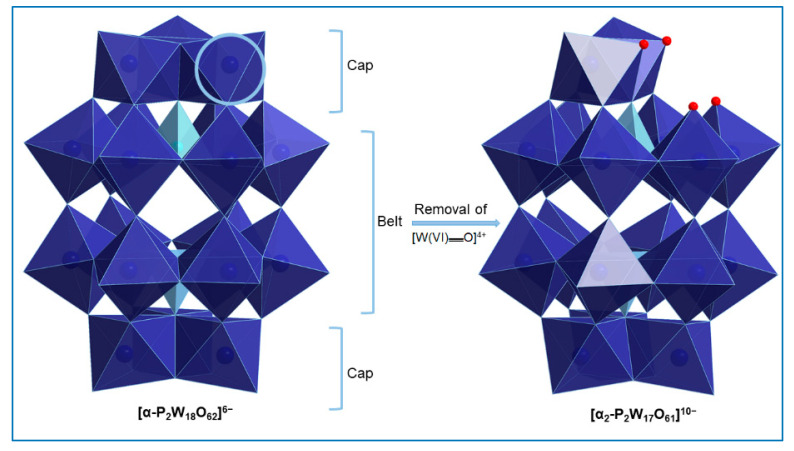
Formation of the [α_2_-P_2_W_17_O_61_]^10−^ isomer by removal of a [W(VI)══O]^4+^ unit from the “cap” segment of the parent [α_-_P_2_W_18_O_62_]^6−^ anion. Color code: WO_6_ blue octahedra, PO_4_ sky blue tetrahedra, O red spheres.

The X-ray structure of **1** reveals that two erbium ions are sandwiched between two monovacant, lacunary Wells−Dawson polyoxoanion [α_2_-P_2_W_17_O_61_]^10−^ units. The compound crystallizes as the hydrated, mixed DMA-sodium salt (NH_2_Me_2_)_13_Na_3_[{Er(H_2_O)(CH_3_COO)(α_2-_P_2_W_17_O_61_)}_2_]·40 H_2_O (**1**) in the triclinic space group *P*Ī. The crystal data of the polyanion **1** are summarized in Table 1. The anionic component of **1** consists of a dinuclear erbium(III) core, [{Er(H_2_O)(CH_3_COO)}_2_]^4+^, which is sandwiched between two [α_2_-P_2_W_17_O_61_]^10−^ units. In POM **1**, one Er^III^ cation occupies the vacant site of each [α_2_-P_2_W_17_O_61_]^10−^ anion and is coordinated to the four available oxygen atoms of the monolacunary site. The two Er^III^ ions are bridged by acetate groups in the η^1^:η^2^:μ_2_ mode and each Er(III) ion is eight coordinated, adopting a square antiprismatic geometry. The coordination sphere of each Er(III) ion is completed by three oxo ligands (from two acetate ions), four oxo ligands (from four coordination sites of the monolacunary ligand [α_2_-P_2_W_17_O_61_]^10−^) and one water molecule (Figure 1). Notably, the formation of the [α_2_-P_2_W_17_O_61_]^10−^ in this structure suggests that the trilacunary precursor [α-P_2_W_15_O_56_]^12−^ rearranges to the monolacunary ligand [α_2_-P_2_W_17_O_61_]^10−^ during the course of the reaction. Such transformations have also been observed in previous work [14]. Concerning the isomerization, the two isomers [α_1-_P_2_W_17_O_61_]^10−^ and [α_2-_P_2_W_17_O_61_]^10−^ of the monolacunary derivative [α_-_P_2_W_17_O_61_]^10−^ can be prepared by removal of a [W(VI)══O]^4+^ unit from the “belt” or the “cap” segments of the parent [α-P_2_W_18_O_62_]^6−^ anion, respectively. These monovacant species are known to act as tetradentate ligands particularly for rare earth metal cations with four strongly basic O donor atoms directed at the void created by the removal of the [W(VI)══O]^4+^ group (Figure 2).

### 2.3. PXRD Analysis

PXRD was used to confirm the identity and phase purity of the crystalline product. The experimental PXRD pattern of the bulk material was almost identical to the simulated PXRD pattern from the solved crystal structure of **1**. The slight shift in peak positions is due to the fact that the PXRD measurement was performed at room temperature during which removal of some crystal water might cause the change in the unit cell parameters. The consistence between the experimental PXRD pattern and the simulated one validates the fact that the crystal structure of **1** is truly representative of the bulk material **1** (Figure S1).

### 2.4. Vibrational Spectroscopy

The FTIR spectra of the isolated POM **1** and of K_10_[α-2-P_2_W_17_O_61_] {P_2_W_17_} show similar characteristic asymmetric vibrations in the region of 1100–400 cm^−1^, which represent the “fingerprint region” of the inorganic POM ligands. However, the comparison of the whole spectral range clearly indicates that **1** had an organic moiety in the form of acetate bridges and dimethyl ammonium cations [NH_2_Me_2_]^+^ (DMA) as organic countercations. Three similar peaks of ***ν***_as_(P–O) were observed at 1085, 1057 and 1017 cm^−1^ in **1** and at 1082, 1048 and 1015 cm^−1^ in {P_2_W_17_}. The peaks at 943 and 919 cm^−1^ in **1** and at 939 and 915 cm^−1^ in {P_2_W_17_} could be assigned to terminal ***ν***_as_(W══O_t_) vibration. The frequencies in the range of 888–850 cm^−1^ and 885–850 cm^−1^ corresponded to corner-sharing ***ν***_as_(W–O_b_–W) of **1** and {P_2_W_17_} respectively. The peaks at 801–708 cm^−1^ in **1** and at 808–731 cm^−1^ in {P_2_W_17_} could be attributed to edge-sharing ***ν***_as_(W–O_c_–W). All these bands are considered as pure vibrations of POM ligands [36]. With respect to the organic moiety in **1**, the resonances at 1557–1464 cm^−1^ and 1438–1349 cm^−1^ appeared due to ***ν***_as_(C══O) and ***ν***_as_(C–O) stretching vibrations of the acetate ligands, which are bridged to the Er(III) cations in a η^1^:η^2^:μ_2_ mode. The adjacent peak around 1600 cm^−1^ can be attributed to the δ(O–H) of the lattice water molecules. In addition, the ***ν***(C–N) stretching band and the δ(C–H) bending vibration were found at 1464 cm^−1^ and 1414 cm^−1^, respectively, suggesting the existence of dimethyl ammonium cations [NH_2_Me_2_]^+^ as organic countercations. The broad band at around 3600–3200 cm^−1^ in **1** reflect stretching vibrations of lattice and coordinated water molecules (Figure S2).

### 2.5. Thermogravimetric Analysis

The thermal decomposition processes for **1** was investigated under an N_2_ atmosphere from room temperature to 800 °C (Figure S3). The TG curve displays a three-step weight loss (up to a total of 15.5%) associated with the loss of 10 water molecules adsorbed from the synthetic solution onto the crystal surface, 30 crystal water molecules, coordinated water molecules, acetate ligands and dimethyl ammonium cations. The first weight loss of ca. 7.5% ranging from room temperature to 250 °C was attributed to the loss of 40 water molecules and coordination water molecules (at higher temperatures). The second step weight loss (250–650 °C) of 8% could be approximately assigned to the decomposition of 13 [NH_2_Me_2_]^+^ cation groups and two acetate ligands in the compound.

### 2.6. Mass Spectrometry

**Figure 3:**
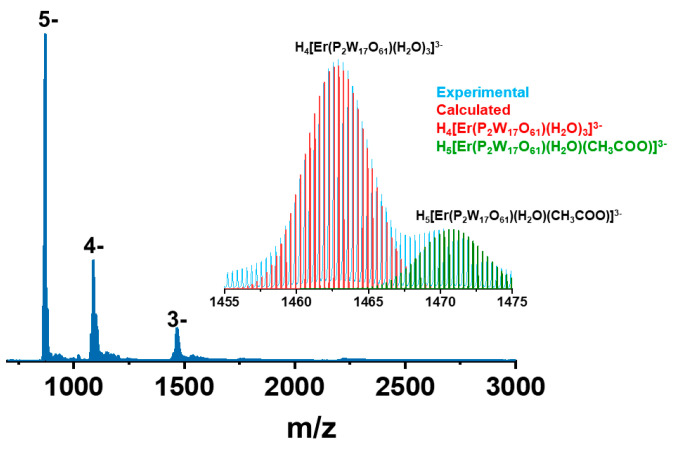
Negative ion electrospray ionization mass spectrometry (ESI MS) of **1** in a water/ACN mixture. The 3- region is expanded in the inset and two peaks are compared with their calculated isotope pattern.

Solution and gas phase stability of the POM **1** was studied using electrospray ionization mass spectrometry (ESI MS). A Waters’ Synapt High Definition Mass Spectrometr (HDMS) was used for this purpose and the samples were measured in the negative ion mode (see experimental details for optimized parameters in SI). A few crystals were dissolved in water diluted with acetonitrile (ACN) at the 50:50 (***v***/***v***) ratio. While the crystal structure revealed a dimeric structure bonded through Er^III^ ions and acetate ligands, they fall apart during the electrospray condition. Several instrumental parameters were optimized to identify the intact ion but even at the lowest possible capillary voltage of 800 V, the two units were separated. The reason could be lesser stability of the intact molecule in the water/ACN mixture. To overcome this issue, the sample was dissolved in the mixture of water and 0.5 M LiOAc/HOAc buffer (pH 4.8) at the 50:50 (***v***/***v***) ratio but the intact ion was not found (Figure S4). About 20 µL of formic acid was added to exchange the alkali metal counter ions with H^+^ for the exact identification of the ion. The resulting mass spectrum is shown in Figure 3. Addition of further formic acid stabilized the 3- charge state with slight contribution from the 2- as shown in Figure S5. The 3- charge state was expanded in the inset. The two peaks corresponded to H_5_[Er(P_2_W_17_O_61_)(H_2_O)(CH_3_COO)]^3−^ at ***m***/***z*** 1470 and H_4_[Er(P_2_W_17_O_61_)(H_2_O)_3_]^3−^ at ***m***/***z*** 1463, respectively. Most of the water molecules seen in the crystal structure were lost during the electrospray process. One of the reasons for the fragmentation could be the exchange of acetate ligands by aqua ligands in aqueous medium during electrospray leading to monomeric units. The acetate ligands connected to the rare earth cation are labile enough to be potentially exchangeable with solvent water molecules or any organic substrates present in the solution [37,38]. The attempt to observe the intact ion in 1 M of LiOAc/HOAc buffer (pH 4.8) was not possible, since, even the dilute LiOAc/HOAc buffer (water and 0.5 M of LiOAc/HOAc buffer (pH 4.8) at a 50:50 (***v***/***v***) ratio) caused the contamination/blockage of the mass spectrometry chamber. The other and most probable reason could be coulomb repulsion between two highly negatively charged cores in very close proximity leading to the fragmentation during electrospray.

### 2.7. UV-Vis Spectroscopy

A UV-Vis spectroscopy study was performed on **1** in order to check its photophysical properties. The UV spectrum of **1** in the range of 500–200 nm exhibited two strong absorption maxima at 230 nm and 280 nm (Figure S6). The former higher energy absorption band can be assigned to the pπ–dπ charge-transfer transitions of the O_t_→W bonds whereas the latter lower energy absorption band is attributed to the pπ–dπ charge-transfer transitions of the O_b(c)_→W bonds.

### 2.8. Emission Spectroscopy

Room temperature photoluminescence experiments were performed on **1** and on K_10_[α*_2_*-P_2_W_17_O_61_]·20H_2_O {α_2_-P_2_W_17_}. Apart from the intensity, similar emission profiles were observed with visible peaks at 301, 402, 459, 485, 525 and 603 nm, when **1** and {α_2_-P_2_W_17_} were excited at 250 nm (Figure S7). This result suggests that both compounds exhibited tungstate emission. The electronic nature of the incorporated erbium ions had almost no effect on the luminescence efficiency.

### 2.9. Electrochemical Characterization

Electron transfer phenomena with POMs are very often concomitant with proton transfer, which may result in drastic pH changes in the vicinity of the electrode surface if the medium is not buffered. The stability and the redox features of POMs being strongly dependent on the pH, the use of buffers is inevitable in order to prevent either the decay of POMs or certain behaviors related to non-controlled pH shifts. Further, based on the information from MS studies and having the knowledge that most often the acetate-bridged complexes tend to disintegrate in aqueous media on ligand substitution with water ligands, we decided to perform cyclic voltammetry (CV) in 1 M of LiOAc + HOAc buffer in order to reduce the possibility of fragmentation to monomeric units in excess of acetate ligands.

**Figure 4:**
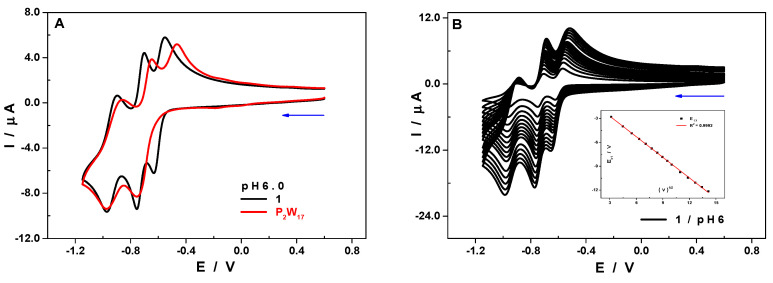
(**A**) Cyclic voltammetry (CV) of **1** (black) and P_2_W_17_ (red) recorded at a scan rate of 50 mV.s^−1^. (**B**) CV of **1**, recorded at scan rates varying from 200 to 10 mV.s^−1^. The insert shows the variation of Ec_1_ as a function of the square root of the scan rate. The CV are obtained in 1.0 M of LiOAc + HOAC/pH 6.0. POM concentrations: [1] = 0.20 mM, [P_2_W_17_] = 0.4 mM. Working electrode: glassy carbon (GC); counter electrode: Pt gauze; reference electrode: SCE.

The superposition of the CV of **1** and of the ligand P_2_W_17_ recorded in the same experimental conditions, 1.0 M of LiOAc + HOAc/pH 6.0 (Figure 4A, where the concentrations have been adjusted in order to facilitate the comparison), revealed some singularities attributable to the presence of the Er^III^ cation. The first reduction step of the compound **1** is clearly distinct from the second one, having a reduction peak potential of Ec_1_ = −0.63 V vs. saturated calomel electrode (SCE), and the potential difference between the two equals ΔE = Ec_1_ − Ec_2_ = 0.13 V (see Table 2). The CV of the P_2_W_17_ ligand shows that the first two bielectronic reduction waves [39] had merged into a single step whose peak potential coincided with that of the second reduction wave of the compound **1**, −0.76 V vs. SCE. The ratio between the peak currents of the waves allows one to infer that the three redox steps of **1** imply the transfer of four electrons each. The linear dependency of the peak current on the square root of the scan rate revealed that the electron transfer process was diffusion-controlled (Figure 4B). The same behavior was also observed at the other pH values (Figure S8).

**Figure 5:**
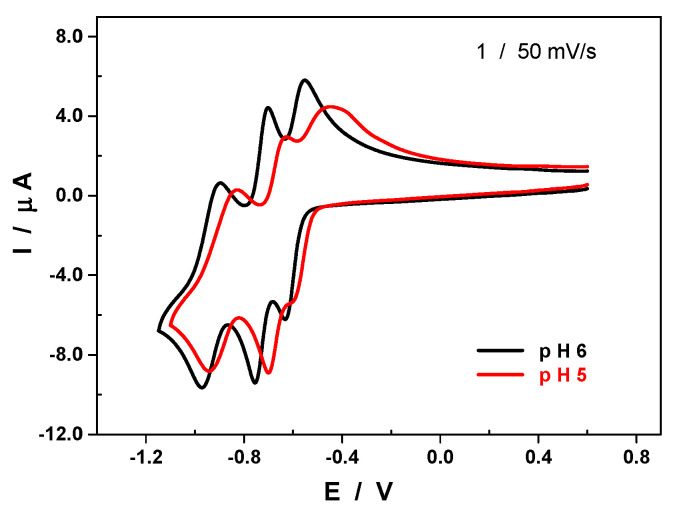
CV of **1** recorded in 1.0 M of LiOAc + HOAC/pH 6.0 (black) and pH 5.0 (red) at a scan rate of 50 mV.s^−1^. POM concentration: [1] = 0.20 mM. Working electrode: GC; counter electrode: Pt gauze; reference electrode: SCE.

When the pH of the electrolyte was varied while keeping the buffer concentration constant, a trend was observed where the peak potentials shifted towards the anodic side of the scale upon decreasing the pH, as expected [40,41]. Additionally, the distinction between the first two reduction steps became less obvious (Figure 5). This behavior indicates not just a marked alkaline character, but also that the electron transfer is concomitant with proton exchange. A quick assessment of the variation of the reduction peak potential of the first and of the second waves as a function of the pH indicates that the number of protons exchanged equals that of the electrons transferred (Figure S9). Having said that, a more accurate study would require a broader pH range to be considered.

The CV of **1** recorded in 0.1 M of LiCH_3_CO_2_ + CH_3_CO_2_H/pH 6.0 did not show any evolution with time. If an evolution happened to have been observed, it could be attributed to a change from a dimeric into a monomeric species, but this was not the case. Even in a medium with a much lower ionic strength (0.1 M of LiCH_3_CO_2_ + CH_3_CO_2_H/pH 6.0), in which the dimer is expected to decay, there were no major changes in the CV of **1**, which exhibited the same features as the one recorded at a higher ionic strength (1.0 M of LiCH_3_CO_2_ + CH_3_CO_2_H/pH 6.0), and it remained stable with time (see Figure S10). We may infer that the conversion of the dimer into the monomer happens during MS measurements, as molecules are rapidly desolvated and converted to the gas phase after high-energy collisions under vacuum during MS measurements. Most likely, the gaseous charged structures observed might not resemble the solution structures. Bearing in mind that CV is a softer technique compared to mass spectrometry to analyze the integrity of POM structures in solution, therefore it is expected that the CV obtained in 1 M of LiOAc + HOAc buffer is of the intact molecule in solution.

**Figure 6:**
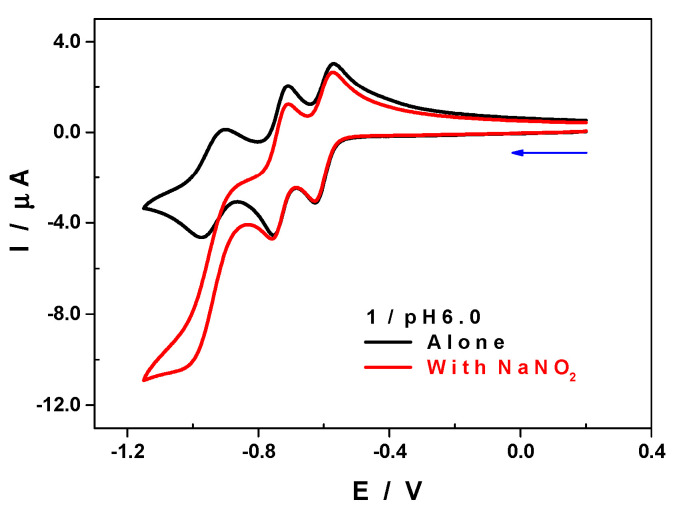
CV of **1** recorded in 1.0 M of LiOAc + HOAc/pH 6.0, in the absence (black) and in the presence (red) of NaNO_2_ at a scan rate of 10 mV.s^−1^. Concentrations: [1] = 0.20 mM, [NaNO_2_] = 10 mM. Working electrode: GC; counter electrode: Pt gauze; reference electrode: SCE.

The reduction of the nitrite ion is a classical test to evaluate the electrocatalytic performance of POMs [42,43]. It was carried out in 1.0 M of LiOAc + HOAc at pH 6, a medium in which the interference from the H.E.R (hydrogen evolution reaction) is negligible and the predominant species of the substrate is the ionic form NO_2_^−^ (HNO_2_/NO_2_^−^; pK_a_ = 3.2). Figure 6 shows the CV of **1** in the absence and in the presence of a large excess of nitrite ([NaNO_2_]/[1] = 50). At a low scan rate, v = 10 mV.s^−1^, there was an electrocatalytic reaction starting right after the second reduction step of **1**, E_onset_ = −0.85 V vs. SCE. This reduction wave was irreversible and its shape was typical of an electrocatalytic process. The comparison of the maximum currents in the absence and in the presence of nitrite ion revealed an increase higher than 350%.

Other than the fact that **1** is more stable in solution than its precursor P_2_W_17_, their behavior towards the reduction of the nitrite ion was rather similar (Figure S11). There was a slight shift of about 50 mV in favor of **1** in the position of the electrocatalytic wave, and the catalytic performance was less pronounced in the case of the compound P_2_W_17_, with an efficiency close to 300% (Figure S11). Despite the stabilization imparted by the Er^3+^ cation to the P_2_W_17_ lacunary structure in **1**, the increase in terms of catalytic efficiency for the reduction of nitrite is rather limited when compared to POMs containing other metallic centers like Cu^2+^ or Ni^2+^ [44,45].

### 2.10. Magnetic Properties

**Figure 7:**
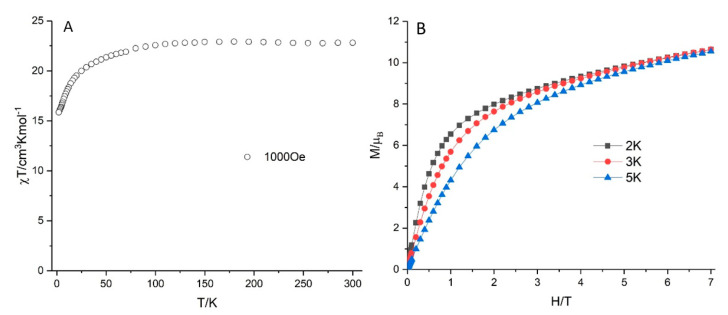
Plot of χT vs. T under 1000 Oe applied DC field (**A**) and plots of M vs. H (**B**) for **1**.

Variable temperature direct-current (DC) magnetic susceptibility of **1** (Figure 7A) was measured on fresh prepared polycrystalline sample under a 1000 Oe applied DC field. The χT value of **1** is 22.81 cm^3^ K mol^−1^ at 300 K, which is close to the expected theoretical values of 23.00 cm^3^ K mol^−1^ for two isolated Er(III) ions (^4^***I***_15/2_, ***S*** = 3/2, ***L*** = 6, ***g**_**J**_* = 6/5 and C = 11.50 cm^3^ K mol^−1^). There is a steady decrease in the χT product on lowering the temperature from 300 to 100 K and a more rapid decrease from 100 to 2 K, reaching to a minimum value of 15.86 cm^3^ K mol^−1^ at 2 K. The decrease is probably caused by the thermal depopulation of the excited m_J_ states of Er(III) as well as the antiferromagnetic intramolecular dipolar interactions between the Er(III) ions. The field dependence of magnetizations for **1** is shown in Figure 7B. The magnetization at 7 T of 10.5 μ_B_ for **1** was close to the expected theoretical values of 9 μ_B_ for two Er(III) ions (^4^***I***_15/2_, ***J*** = 15/2, ***g**_**J**_* = 6/5), which is similar to the previously reported Er_2_ complex [46].** AC susceptibility measurements were also performed in order to investigate the potential SMM behavior (Figure S12). There were no ac signals under a zero applied DC field. However, it did show very weak signals without maxima, under small applied DC fields (1000–4000 Oe); however, this slow relaxation did not indicate that **1** is a SMM. The square-antiprismatic coordination polyhedron of Er(1) (Figure 1B) might at first sight seem favorable for SMM behavior. The oxido oxygens O(9), O(19), O(12) and O(17) formed a well-defined square (O···O 2.710–2.924 Å, O-O-O 87.5–93.3°), with the zenithal angles at Er(1) subtended by the O_4_ centroid and each of the oxygens (58.6–63.4°) all being well above the “magic angle” (54.7°) [47]. However, the rather low symmetry of the other square face of the antiprism, a consequence of the much shorter O···O distance between the two oxygens of the chelating acetate, will not only reduce any barrier to magnetization reversal, but also promote quantum tunneling of the magnetization.

## 3. Experimental Section

The POM ligand, Na_12_[α-P_2_W_15_O_56_]^.^18H_2_O {P_2_W_15_} was synthesized according to the literature methods and was characterized by FTIR spectroscopy [48]. All reactions were carried out under aerobic conditions. All other reagents were purchased commercially and were used without further purification.

### Synthesis

Synthesis procedure for (NH_2_Me_2_)_13_Na_3_[{Er(H_2_O)(CH_3_COO)(α_2-_P_2_W_17_O_61_)}_2_]·40 H_2_O **(1)**

Na_12_[α-P_2_W_15_O_56_]^.^18H_2_O (0.88 g, 0.20 mmol) was dissolved in 20 mL of 1 M of LiOAc/HOAc buffer (pH 4.8). Then 0.30 g (0.65 mmol) of Er(NO_3_)_3_^.^6H_2_O was added, which caused the solution to become light pink. The solution was heated at 90 °C for one hour. After heating, the solution was filtered and 0.05 mL of 1 M of dimethylammonium chloride solution, (CH_3_)_2_NH_2_Cl was added to the clear filtrate. Slow evaporation of the clear solution led to light pink crystals after approximately two weeks, which were isolated by filtration and dried in air. Yield 120 mg. IR (2% KBr pellet, ν/cm^−1^): 1631 (sh), 1560 (s), 1463 (s), 1493 (w) 1432 (w), 1056 (m), 1016 (s), 943 (w), 917 (w) 796 (br), 701 (br), 524 (s), 478 (w). Elemental analysis (%) calculated: W 61.47, P 1.22, Er 3.29, Na 0.68, C 3.54, N 1.79, H 1.88; found: W 61.3, P 1.22, Er 3.67, Na 0.69, C 3.52, N 1.64, H 1.59.

## 4. Conclusions

We isolated a dinuclear erbium(III) substituted sandwich-type POM [{Er(H_2_O)(CH_3_COO)(P_2_W_17_O_61_)}_2_]^16−^ in a simple one pot synthetic procedure and characterized it in the solid as well as in the solution state. The monolacunary Dawson units in polyanion **1** were formed by the transformation of the trilacunary POM precursor {α-P_2_W_15_} into the {α_2_-P_2_W_17_} fragment in the presence of Er^III^ ions. Magnetic studies revealed that **1** demonstrated antiferromagnetic behavior. Mass spectrometry was performed to examine the structure of **1** in the solution/gas phase. The electrochemical properties of **1** were studied by means of cyclic voltammetry in in 1.0 M of LiOAc + HOAC buffer solution as a supporting electrolyte. Our study demonstrated that the acetate-bridged dimer {α_2_-ErP_2_W_17_} type of lanthanoid-containing polyoxometalates could be obtained in the presence of an excess of carboxylic ligands as bridging connectors. Through this synthetic strategy, new novel materials based on lacunary POMs and Ln cations can be designed. In the following work, we will introduce a series of Ln cations to the present system to exploit the novel functional materials.

## Figures and Tables

**Table 1:** Crystallographic data for **1**.

Formula	C_30_H_174_Er_2_N_13_Na_3_O_158_P_4_W_34_
Formula weight/g mol^−1^	10,024.08
Crystal System	triclinic
Space Group	*P* $\bar{1}$
***a***/Å	13.2960(2)
***b***/Å	13.4153(2)
***c***/Å	27.0804(4)
α/°	91.764(1)
β/°	92.500(1)
γ/°	111.721(1)
***U***/Å^3^	4432.70(12)
Z	1
T/K	180(2)
***F***(000)	4454
***D_c_***/Mg m^−3^	3.755
µ(Ga-Kα)/mm^−1^	32.074
Data Measured	77,253
Unique Data	18,640
***R_int_***	0.0553
Data with I ≥ 2σ(I)	15,225
***wR_2_*** (all data)	0.1944
***S*** (all data)	1.040
***R_1_*** [II ≥ 2σ(I)]	0.0641
Parameters/Restraints	1169/70
Biggest diff. peak/hole/eÅ^−3^	3.66/−4.90
CSD number	2,021,556

**Table 2:** Reduction, Ec, and oxidation, Ea, peak potentials (mV vs. SCE) measured from the CV of **1** and of P_2_W_17_ recorded in the experimental conditions specified above.

		Ec_1_	Ec_2_	Ec_3_	Ea_1_	Ea_2_	Ea_3_
**pH 6**	**P_2_W_17_**		−0.76	−0.98	−0.47	−0.65	−0.86
	**1**	−0.63	−0.76	−0.97	−0.55	−0.70	−0.90
**pH 5**	**1**	−0.60	−0.70	−0.94	−0.45	−0.63	−0.83
**pH 4**	**1**	−0.50	−0.65	−0.90	−0.30	−0.43	−0.78

## Supplementary Materials

Infrared spectra, thermogram, UV–vis spectrum, emission spectra and additional cyclic voltammograms, ac magnetic data and mass spectra. As well as crystallographic data in the CIF format.
