# Supplementary material for: Synthesis, Characterization, Electrochemistry, Photoluminescence and Magnetic Properties of a Dinuclear Erbium(III)-Containing Monolacunary Dawson-Type Tungstophosphate: [{Er(H2O)(CH3COO)(P2W17O61)}2]16−
Source: Molecules. 2020 Sep 15;25(18):4229. doi: 10.3390/molecules25184229 (PMC7570939; doi:10.3390/molecules25184229)
Supplement: Supplementary file 1 [file molecules-25-04229-s001.pdf]

Electronic Supplementary Information (ESI)

Synthesis, Characterization, Electrochemistry, Photoluminescence and Magnetic Properties of a Dinuclear Erbium(III)-Containing Monolacunary Dawson-Type Tungstophosphate: $[\{Er(H_2O)(CH_3COO)(P_2W_{17}O_{61})\}_2]^{16-}$

Masooma Ibrahim[1,*], Ananya Baksi[1], Yan Peng[2], Firas Khalil Al-Zeidaneen[2], Israël M. Mbomekallé[3], Pedro de Oliveira[3] and Christopher E. Anson[2]

[1]Institute of Nanotechnology, Karlsruhe Institute of Technology, Hermann von-Helmholtz Platz 1, 76344 Eggenstein-Leopoldshafen, Germany

[2]Institute of Inorganic Chemistry, Karlsruhe Institute of Technology, Engesserstrasse 15, 76131 Karlsruhe, Germany

[3]Equipe d'Electrochimie et de Photo-électrochimie, Institut de Chimie Physique, Université Paris-Saclay, UMR 8000 CNRS, Orsay F-91405, France

Experimental

Materials and methods

The POM ligand, $Na_{12}[\alpha\text{-}P_2W_{15}O_{56}]\cdot 18H_2O$ $\{P_2W_{15}\}$ was synthesized according to the literature methods and was characterized by FTIR spectroscopy [1]. All reactions were carried out under aerobic conditions. All other reagents were commercially purchased and were used without further purification. The crystal structure was measured at 180 K on a STOE STADIVARI diffractometer with a Dectris Eiger2 R 4M detector using Ga-Kα radiation (λ = 1.34143 Å) from a MetalJet2 source at the Institute of Nanotechnology, Karlsruhe Institute of Technology. X-Ray powder diffraction patterns were measured at room temperature using a Stoe STADI-P diffractometer with a Cu-Kα radiation at the Institute of Nanotechnology, Karlsruhe Institute of Technology. Fourier transform IR spectra were measured on a Perkin-Elmer Spectrum One Spectrometer with samples prepared as KBr discs. The following abbreviations were used to describe the peak characteristics: br = broad, sh = shoulder, s = strong, m = medium and w = weak Elemental compositions were determined

by an Inductively Coupled Plasma with Optical Emission Spectroscopy (ICP-OES) at the Institute for Applied Materials (IAM-AWP), Karlsruhe Institute of Technology. Thermogravimetric analysis was performed on a SDT Q 600 device from TA instruments at the Jacobs University, Bremen for 18 mg of sample at a heating rate of 5.00 ℃/min to 800.00 ℃ in an alumina pan under 100mL/min nitrogen flow. A Waters' Synapt HDMS was used for the mass spectrometric analysis. All the samples were measured in the negative ion mode. A few crystals were dissolved in water diluted with acetonitrile (1:1, *v/v*). The optimized parameters are as follows: flow rate: 10 µL/min; capillary voltage: 1.2 kV; cone voltage: 10 V; source offset: 10V; source temperature: 80 ℃; desolvation temperature: 150 ℃; desolvation gas flow: 400 L/h; nebulizer: 2.5 bar.

Crystallography

Data on a single crystal of **1** were collected at 180 K on a STOE STADIVARI diffractometer with a Dectris Eiger2 R 4M detector using Ga-Kα radiation (λ = 1.34143 Å) from a MetalJet2 source at the Institute of Nanotechnology, Karlsruhe Institute of Technology. Semi-empirical absorption corrections were applied using LANA [2].The structure solution was achieved by dual-space direct methods (SHELXT) [3] followed by full-matrix least-squares refinement (SHELXL-2018) [4] within the Olex2 platform (Table 1) [5]. Anisotropic temperature factors were used for all ordered non-H atoms, The H-atoms of the water ligands were refined with geometrical restraints. H-atoms of the acetate ligands and $(Me_2NH_2)^+$ cations were placed in calculated positions; no attempt was made to model the H-atoms of the lattice waters. Disordered $(Me_2NH_2)^+$ cations were mostly refined anisotropically with rigid-bond and geometrical similarity restraints. Similarity restraints were assigned to the thermal parameters of adjacent atoms from different disorder components as required.

Further details of the crystal structures investigation may be obtained from FIZ Karlsruhe, 76344 Eggenstein-Leopoldshafen, Germany, https://www.ccdc.cam.ac.uk/structures/, on quoting the deposition number CSD-2021556

Electrochemical studies

Pure water was obtained with a Milli-Q Integral 5 purification set. All reagents were of high-purity grade and were used as purchased without further purification: HOAc (Glacial, Prolabo Normapur), and LiOAc.2H_2O (Acros Organics). The composition of the various media was as follows: 1.0 M LiOAc + HOAc for pH values 4.0, 5.0 and 6.0. The stability of the different compounds in solution was assessed by cyclic voltammetry.

Electrochemical data were obtained using an EG & G 273 A potentiostat driven by a PC with the M270 software. A divided cell with a standard three-electrode configuration was used for cyclic voltammetry experiments. The reference electrode was a saturated calomel electrode (SCE) and the counter electrode a platinum gauze of large surface area; both electrodes were separated from the bulk electrolyte solution via fritted compartments filled with the same electrolyte. The working electrode was a 3 mm outer diameter glassy carbon (GC) from Mersen, France. The pre-treatment of the electrode before each experiment is adapted from a method described elsewhere [6]. Prior to each experiment, solutions were thoroughly de-aerated for at least 30 min with pure argon. A positive pressure of this gas was maintained during subsequent work. All cyclic voltammograms (CVs) were recorded at a scan rate of 5 mV s^{-1} and potentials are quoted against SCE unless otherwise stated. The polyanion concentrations were 0.2 mM for **1** and 0.4 mM for $[P_2W_{17}O_{61}]^{10-}$. All experiments were performed at room temperature, which is controlled and fixed for the laboratory at 20 ℃. Results were very reproducible from one experiment to the other and slight variations observed over successive runs are rather attributed to the uncertainty associated with the detection limit of our equipment (potentiostat, hardware and software) and not to the working electrode pre-treatment nor to possible fluctuations in temperature.

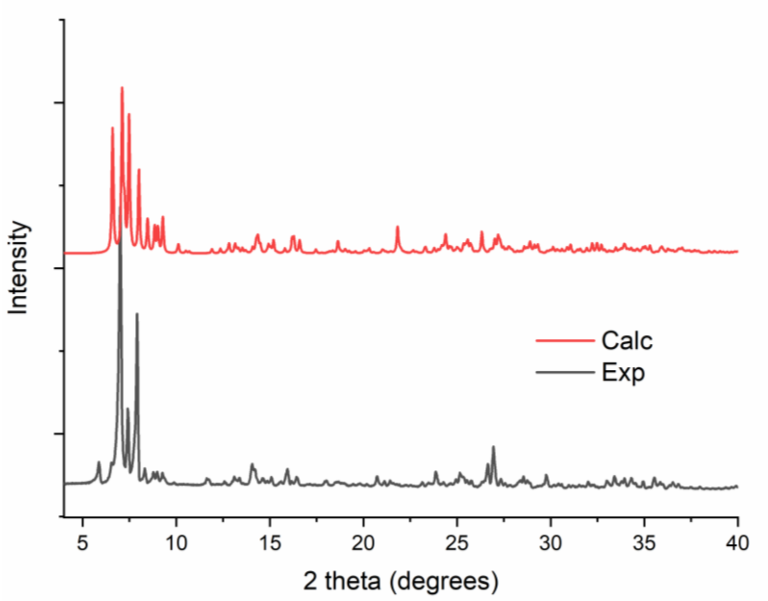

Figure 1. Experimental powder diffraction pattern of **1** (black) and the calculated powder diffraction pattern from the single-crystal X-ray diffraction structure (red).

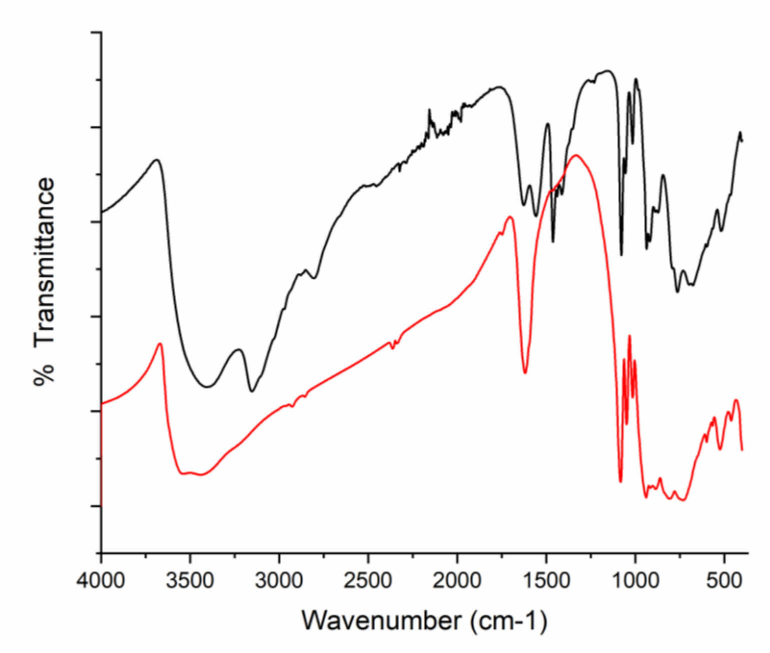

Figure S2. FT-IR spectra of **1** (black) and of $K_{10}[\alpha_2\text{-}P_2W_{17}O_{61}]$ (red).

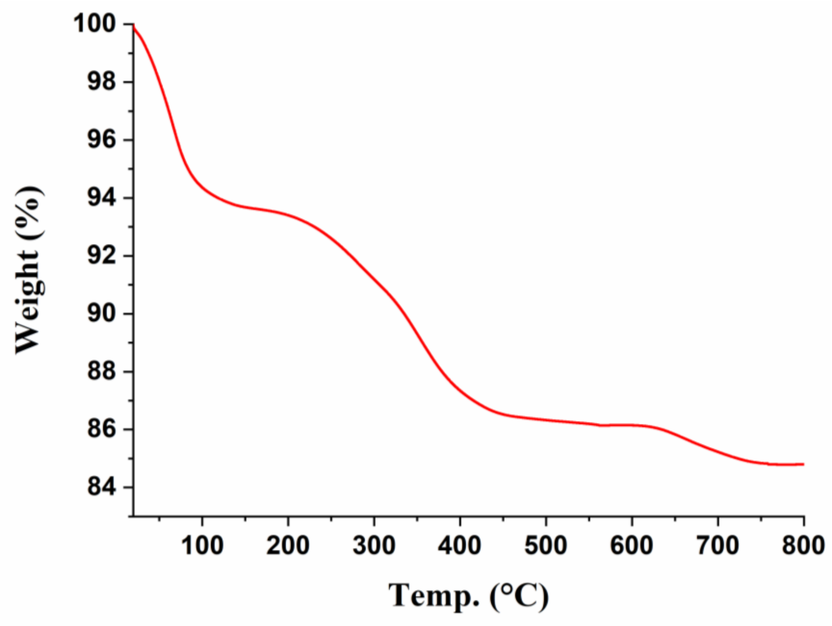

Figure S3. Thermogravimetric analysis (TGA) curve of **1**.

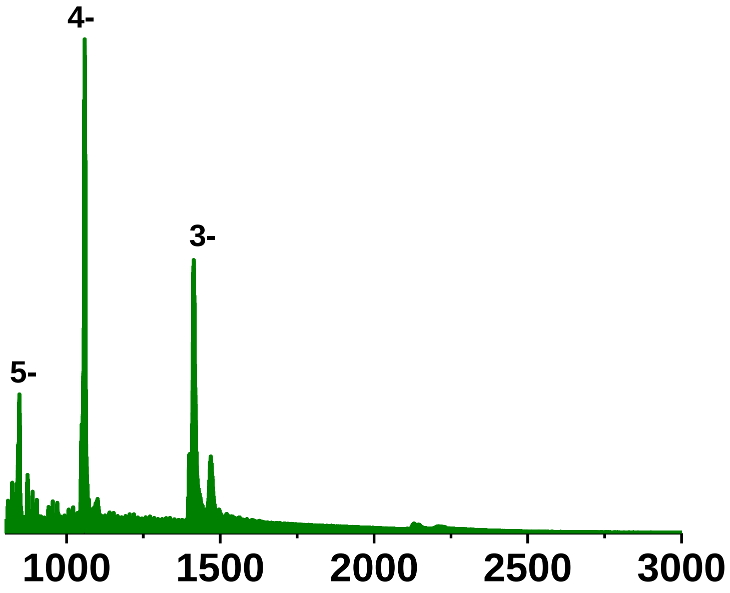

Figure S4. Negative ion ESI MS of **1** in mixture of water and 0.5 M LiOAc/HOAc buffer (pH 4.8) at 50:50 (v/v) showing different charge state of the monomeric fragment (see Figure 3 in the main text for details). Several LiOAC cluster ions were also seen along with the POM ion in the lower mass range.

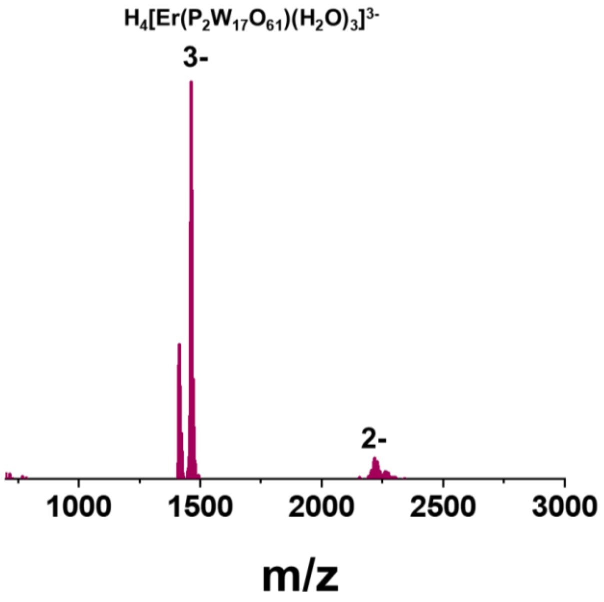

Figure S5. Negative ion ESI MS of **1** in Water/ACN mixture with 40 µL of Formic acid showing predominantly the 3- charge state of the monomeric fragment (see Figure 3 in the main text for details).

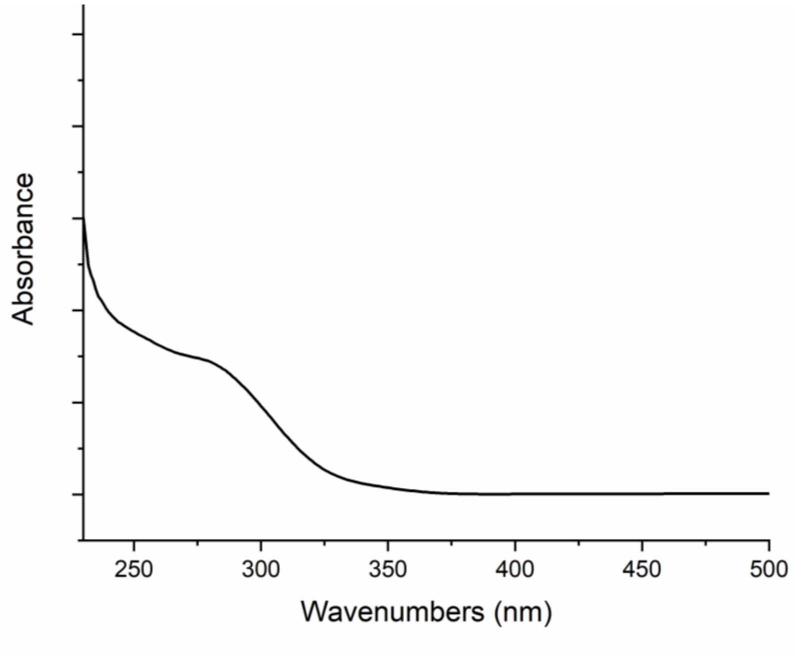

Figure S6. UV-Vis spectrum of **1**.

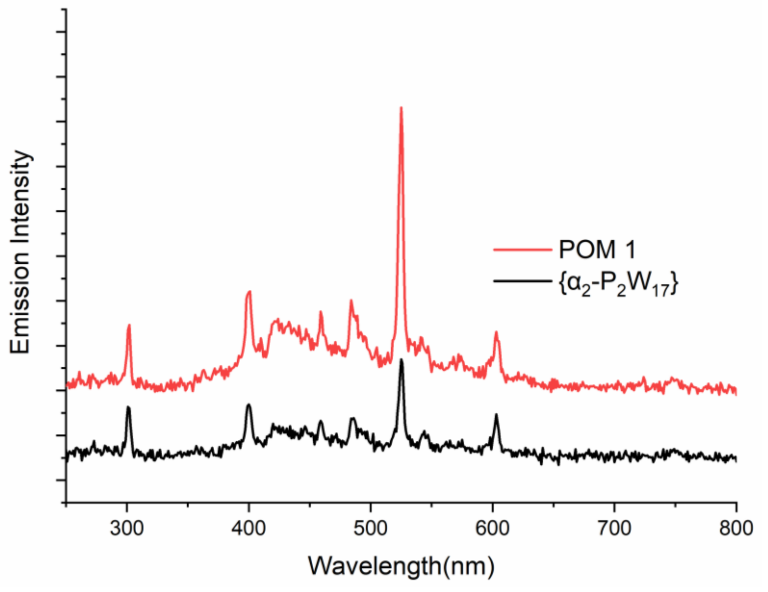

Figure S7. Emission spectra of spectra of **1** (red) and of $K_{10}[\alpha_2\text{-}P_2W_{17}O_{61}]$ (black).

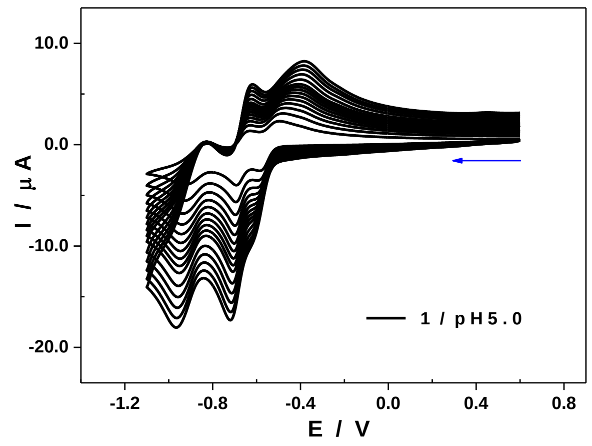

Figure S8. CVs of **1**, recorded at scan rates varying from 200 to 10 mV.s^{-1}. The CVs are obtained in 1.0 M LiOAc + HOAc / pH 5.0. POM concentrations: [**1**] = 0.20 mM. Working electrode: GC; counter electrode: Pt gauze; reference electrode: SCE.

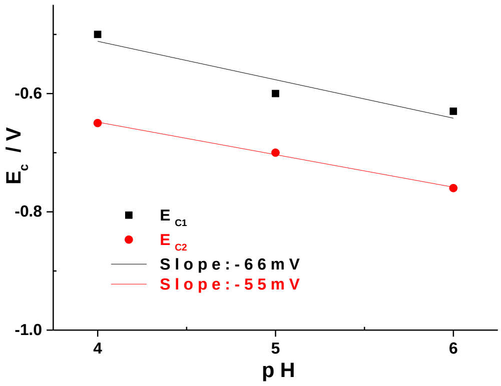

Figure S9. Variation of Ec_1 and of Ec_2 as a function of the pH.

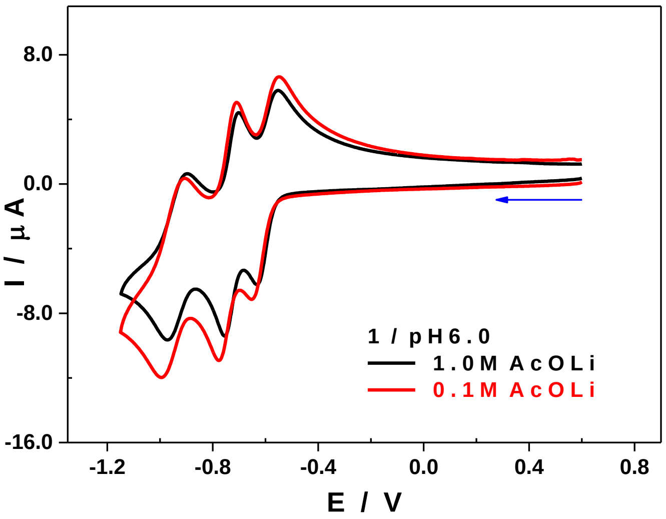

Figure S10. CVs of **1** in 1.0 M $LiCH_3CO_2$ + CH_3CO_2H / pH 6.0 (black) and in 0.1 M $LiCH_3CO_2$ + CH_3CO_2H / pH 6.0 (red) recorded at a scan rate of 50 mV.s^{-1}. POM concentration: 0.20 mM, Working electrode: GC; counter electrode: Pt gauze; reference electrode: SCE.

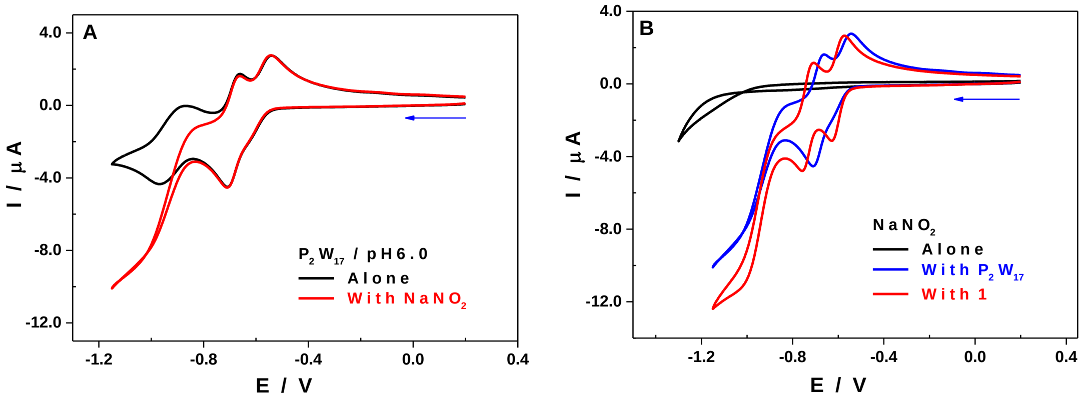

Figure S11. CVs recorded in 1.0 M LiOAc + HOAc / pH 6.0, at a scan rate of 10 mV.s^{-1}. **(A)** $\mathbf{P_2W_{17}}$ in the absence (black) and in presence (red) of $NaNO_2$; concentrations: [$\mathbf{P_2W_{17}}$] = 0.4 mM, [$\mathbf{NaNO_2}$] = 20 mM. **(B)** $\mathbf{NaNO_2}$, alone (black), and either in the presence of $\mathbf{P_2W_{17}}$ (blue) or of **1** (red); concentrations: [$\mathbf{NaNO_2}$] = 20 mM; [**POM**]/[$\mathbf{NaNO_2}$] =50. Working electrode: GC; counter electrode: Pt gauze; reference electrode: SCE.

Magnetism

Dc magnetic susceptibility data (2–300 K) were collected on powdered samples restrained in eicosane and placed in sealed plastic bag using a Quantum Design model MPMS-XL SQUID magnetometer under an applied field of 1000 Oe. Magnetization data were collected between 0 and 7 T at 2, 3 and 5 K. Ac susceptibility measurements were performed with an oscillating field of 3 Oe and ac frequencies ranging from 1 to 1500 Hz. All data were corrected for the contribution of the sample holder.

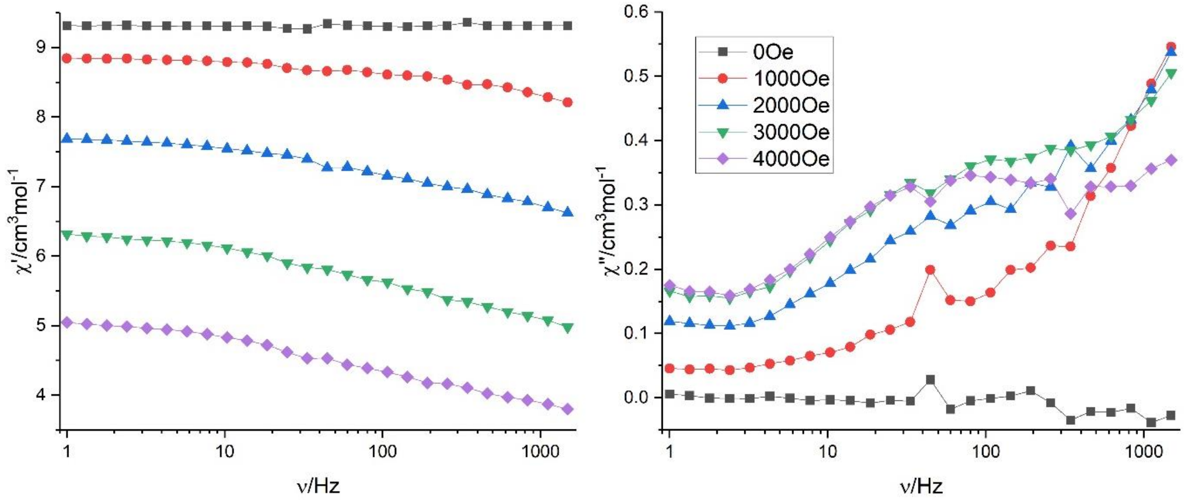

Figure S12. Frequency dependence of in phase (χ') and out-of-phase (χ'') for **1** under the indicated fields at 2 K. The solid lines are guides for the eyes.

References

1. Finke, R. G.; Droege, M. W.; Domaille, P. J. Rational Syntheses, Characterization, Two-Dimensional Tungsten-183 NMR, and Properties of Tungstometallophosphates $P_2W_{18}M_4(H_2O)_2O_{68}^{10-}$ and $P_4W_{30}M_4(H_2O)_2O_{112}^{16-}$ (M = Cobalt, Copper, Zinc). *Inorg. Chem.* **1987**, *26*, 3886–3896.
2. STOE LANA, absorption correction by scaling of reflection intensities. J. Koziskova, F. Hahn, J. Richter, J. Kozisek, *Acta Chimica Slovaca*, **2016**, *9*, 136–140.
3. Sheldrick, G. M. *Acta Crystallogr.*, *Sect. A*: *Found. Adv*. **2015**, *71*, 3–8.
4. Sheldrick, G. M. *Acta Crystallogr.*, *Sect. C*: *Struct. Chem*. **2015**, *71*, 3–8.
5. Dolomanov, O. V.; Bourhis, L. J.; Gildea, R. J.; Howard, J. A. K.; Puschmann, H. *J. Appl. Crystallogr*., **2009**, *42*, 339–341.
6. Vilà, N.; Aparicio, P. a.; Sécheresse, F.; Poblet, J.M.; López, X.; Mbomekallé, I.M. Electrochemical behavior of α_1/α_2-$[Fe(H_2O)P_2W_{17}O_{61}]^{7-}$ isomers in solution: Experimental and DFT studies. *Inorg. Chem*. **2012**, *51*, 6129–6138.
